# Dermoscopic Features of Mycosis Fungoides and Its Variants in Patients with Skin of Color: A Retrospective Analysis

**DOI:** 10.5826/dpc.1103a48

**Published:** 2021-05-20

**Authors:** Mio Nakamura, Tomas Huerta, Kendrick Williams, Alexandra C. Hristov, Trilokraj Tejasvi

**Affiliations:** 1 Department of Dermatology, University of Michigan, Ann Arbor, Michigan, USA; 2 University of Michigan Medical School, Ann Arbor, Michigan, USA; 3 Department of Pathology, University of Michigan, Ann Arbor, Michigan, USA; 4 Ann Arbor Veterans Health Center, Ann Arbor, Michigan, USA

**Keywords:** cutaneous T-cell lymphoma, mycosis fungoides, dermoscopy, skin of color

## Abstract

**Background:**

Mycosis fungoides (MF) is a cutaneous T-cell lymphoma that disproportionately affects people with skin of color and is difficult to diagnose.

**Objective:**

This study characterized the dermoscopic features of MF and its subtypes in patients with skin of color.

**Methods:**

Dermoscopic images of patients with skin of color seen at the cutaneous T-cell lymphoma clinic at Michigan Medicine Dermatology between 2018 and 2019 were reviewed. Specific dermoscopic features were identified and summarized for each subtype of MF.

**Results:**

A total of 33 dermoscopic images from 11 patients with skin of color were reviewed. Four patients had classic MF (18 dermoscopic images), 4 had hypopigmented MF (9 dermoscopic images), 1 had folliculotropic MF (4 dermoscopic images), and 2 had verrucous MF (2 dermoscopic images). Classic MF was characterized by striking pigmentary change, thick black lines, white rosettes, and geometric white lines. Hypopigmented MF was characterized by the loss of the patient’s natural pigment network. In folliculotropic MF, follicular plugging and hyperpigmented to violaceous perifollicular halos were observed. In verrucous MF, large, yellow-gray amorphous structures with yellow-gray ridges and comedo-like openings were observed within hyperkeratotic areas. Overall, vessel morphology was difficult to discern on dermoscopy.

**Conclusions:**

Dermoscopic features of MF in patients with skin of color are predominantly characterized by striking pigmentary alteration. Vessel morphology is not a reliable diagnostic feature. As patients with MF and skin of color have a worse prognosis than light-skinned individuals, a better understanding of dermoscopic features may aid in early diagnosis and improve outcomes in this group.

## Introduction

Mycosis fungoides (MF) is the most prevalent type of cutaneous T-cell lymphoma (CTCL) [1]. It disproportionately affects people with skin of color (SOC), who have worse outcomes [2–4]. The hallmark of classic MF in SOC patients is the presence of polychromatic patches and plaques, with hypo- and hyperpigmentation and erythema. There are several subtypes of MF, including hypopigmented, folliculotropic, and verrucous MF [5]. Early stage MF can clinically resemble dermatoses such as psoriasis and atopic dermatitis, which may lead to a delay in diagnosis. Dermoscopy can be used to distinguish MF from such entities. Studies have described dermoscopic features of early stage MF, including fine, short, linear vessels and orange-yellow patchy areas [6–8]. A limited number of studies have described dermoscopy findings of folliculotropic MF, including “perifollicular erythema surrounding comedo-like lesions, white structureless areas replacing lost hair follicles, and fine short linear, glomerular, and dotted vessels” [9,10]. Dermoscopic features of the other subtypes of MF are not well described. Furthermore, no studies to date have described dermoscopic features of MF specifically in SOC patients.

In this study, we characterized the dermoscopic features of MF and its subtypes in SOC patients. We hypothesized that the dermoscopic features of MF in SOC patients are unique due to the greater presence of the chromophore melanin. In SOC, melanosomes are larger, more abundant throughout the epidermis, and singly dispersed from melanocytes to keratinocytes. The keratinocyte layers are also thicker in SOC than in lighter skin. These differences pose a challenge to appreciating vessel patterns in skin lesions of SOC patients.

## Methods

This was a single-center, retrospective review of dermoscopic images obtained from patients presenting to the CTCL Clinic at Michigan Medicine Dermatology between 2018 and 2019. Images of all patients with SOC (Fitzpatrick skin type IV-VI) who gave verbal consent to have dermoscopic images obtained were included. Each dermoscopic image was retrospectively and independently reviewed by four of the authors (MN, TH, KW, and TT), and specific dermoscopic features were identified. Any discrepancies were resolved by discussion. Skin biopsy slides for histopathologic correlation were reviewed for selected cases. This study was deemed exempt from the need to obtain the patients’ informed consent by the University of Michigan Institutional Review Board (HUM00155110).

## Results

The study included a total of 33 dermoscopic images from 11 SOC patients. Four patients had classic MF (18 dermoscopic images), 4 had hypopigmented MF (9 dermoscopic images), 1 had folliculotropic MF (4 dermoscopic images), and 2 had verrucous MF (2 dermoscopic images).

**Figure 1:**
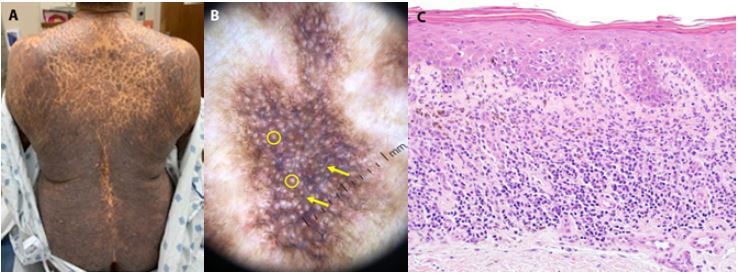
(A) Classic MF was characterized by (B) striking pigmentary change, including a pseudonetwork of brown-gray clods and dots, as well as thick black lines and geometric white lines (yellow arrows) surrounding structureless zones interrupted by prominent eccrine duct openings and white rosettes (yellow circles). (C) Histological analysis revealed an atypical, epidermotropic T-cell infiltrate with a band-like distribution in the superficial dermis and pigment incontinence (H&E, ×200).

Classic MF (Figure 1A) was characterized dermoscopically by striking pigmentary changes, including the formation of pseudonetworks made up of dark brown to gray or black clods and dots. There were thick black lines overlying erythematous to whitish pink, structureless zones, interrupted by prominent eccrine duct openings and white rosettes (Figure 1B). Geometric white lines (Figure 1B) were also seen. Histopathologically, an atypical, epidermotropic T-cell infiltrate with a band-like distribution in the superficial dermis and pigment incontinence were observed (Figure 1C).

**Figure 2:**
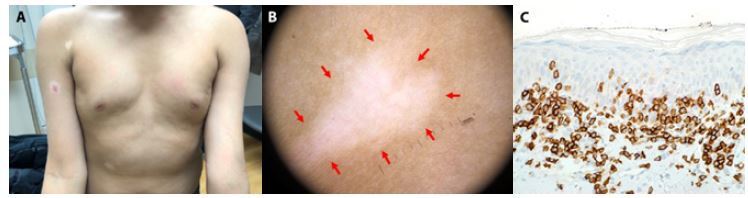
(A) Hypopigmented MF was characterized by (B) patchy, amorphous white-pink areas and loss of the patient’s natural pigment network (outlined by red arrows). (C) Histological analysis demonstrated an atypical lymphoid infiltrate that tagged the dermal-epidermal junction, showed some upward migration in the epidermis, and formed a band-like infiltrate in the dermis. These atypical lymphoid cells expressed CD8 (×400).

Hypopigmented MF (Figure 2A) was characterized by patchy, amorphous, white-pink areas with reduction or loss of the patient’s natural pigment network (Figure 2B). Analysis of biopsy specimens demonstrated an atypical lymphoid infiltrate tagging the dermal-epidermal junction, showing some upward migration in the epidermis, and forming a band-like infiltrate in the dermis. These atypical lymphoid cells expressed CD8 (Figure 2C).

**Figure 3:**
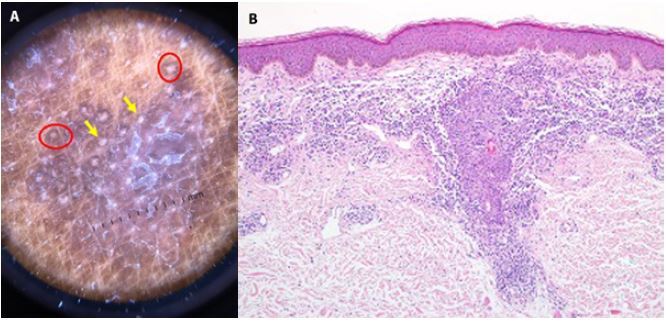
Folliculotropic MF was characterized by (A) follicular plugging (yellow arrows), perifollicular scale, and hyperpigmented to violaceous perifollicular halos (red circles). (B) Histological analysis demonstrated a band-like lymphoid infiltrate in the superficial dermis and intrafollicular atypical T-cells (H&E, ×100).

In folliculotropic MF, dermoscopy showed follicular plugging, perifollicular scale, and hyperpigmented to violaceous perifollicular halos (Figure 3A). On histopathological analysis, a band-like lymphoid infiltrate in the superficial dermis and intrafollicular atypical T-cells were observed (Figure 3B).

**Figure 4:**
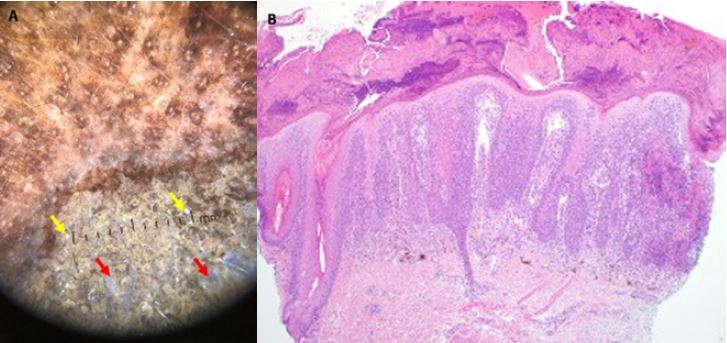
Verrucous MF was characterized by (A) large, multicolored amorphous structures with yellow-gray ridges (red arrows) and comedo-like openings (yellow arrows). (B) Histological analysis showed marked epidermal hyperplasia and hyperkeratosis with an associated epidermotropic, atypical T-cell infiltrate and Pautrier microbascesses (H&E, ×40).

In verrucous MF, large, multicolored amorphous structures with yellow-gray ridges and comedo-like openings mimicking seborrheic keratosis were observed within hyperkeratotic areas (Figure 4A). Histological analysis showed marked epidermal hyperplasia and hyperkeratosis with an associated epidermotropic, atypical T-cell infiltrate and Pautrier microabscesses (Figure 4B). Vessel morphology was difficult to discern on dermoscopy, and the pigmentary alterations described above predominated. Histopathologically, vessels in the upper dermis were obscured by the heavy, atypical lymphocytic infiltrate and pigment incontinence.

## Discussion

MF, especially early stage MF, can be difficult to diagnose; the median time from symptom onset to diagnosis is 4–6 years [11–13]. Although the overall prognosis of early stage MF is favorable, with a median survival of more than 20 years, African-American patients with MF have poorer survival than white patients, even after accounting for disease characteristics, socioeconomic factors, and types of treatment; this finding suggests that there are underlying differences in the biology of the disease between patients with SOC and light skin [14].

Although only a few, small-scale studies have tested dermoscopy in the diagnosis of MF, it seems that dermoscopy can improve the diagnostic accuracy of classic MF [15]. Vessel morphology such as spermatozoa-like, fine, short linear vessels were found more often in MF than in inflammatory dermatoses such as psoriasis and dermatitis, which commonly display dotted vessels [6]. However, previous studies have only described dermoscopic features in light skin. In SOC, vessel morphology is difficult to distinguish due to the greater number of melanosomes and other properties that prevent the dermoscope’s light from penetrating past the basal epidermal layer. This study characterized dermoscopic features of MF in SOC patients, and confirmed that vessel morphology is often unable to be appreciated. This was reflected by the histopathologic findings of vessels obscured by the heavy lymphoid infiltrate along with notable pigment incontinence in the upper dermis. Instead, MF in SOC is characterized by striking pigmentary alteration.

In classic MF, a pigment pseudonetwork consisting of brown-gray clods and dots was present. Thick black lines surrounding structureless areas with rosettes and geometric white lines were also prominent. Superposition of these changes resulted in foci reminiscent of the blue-white veil often described in dermoscopy of melanoma. These features should help one differentiate MF from other papulosquamous disorders. Psoriasis in SOC exhibits dotted vessels under dermoscopy, perhaps due to a thinning of the suprapapillary plates [7]. Irregularly distributed vessels with brown-gray clods are seen in acute eczema [6].

Hypopigmented MF is characterized by loss of the patient’s natural pigment network. Although the cause of hypopigmentation is not known, it is thought to be due to the infiltration of CD8+ cytotoxic T-cells, which damage the melanocytes; this phenomenon has also been described in inflammatory vitiligo [16]. The hypopigmentation in vitiligo under dermoscopy demonstrates a well-defined border, satellite areas, micro-Koebner phenomena and leucotrichia [17]. Clinically, the differential diagnosis includes nevus depigmentosus and idiopathic guttate hypomelanosis. In the former, the dermoscopy features include retention of the pigment network within the hypopigmented area, while in the latter, a well-defined hypopigmented clod with an accentuated pigment network in the periphery can be appreciated. These features may help discern these benign conditions from hypopigmented MF.

Folliculotropic MF was characterized by follicular plugging along with perifollicular hyperpigmentation; the latter feature has not been described in light-skinned individuals. This study also highlights the first dermoscopic description of verrucous MF in SOC: Multicolored amorphous structures with yellow-gray ridges and comedo-like openings mimicking seborrheic keratoses were observed within hyperkeratotic areas.

This study is limited by its single-center, retrospective nature and small sample size. Larger studies are needed to better delineate dermoscopic features of MF in SOC patients. However, this is the first report to date to describe dermoscopic features of MF and its variants in SOC.

## Conclusions

MF in SOC patients is distinct from MF in patients with light skin, and it is characterized by striking pigmentary changes. Dermoscopic features such as rosettes and geometric white lines are unique to MF in SOC patients, while vessel morphology is not a reliable diagnostic feature. Given that SOC patients with MF have worse prognosis than light-skinned individuals, a better understanding of the dermoscopic features may aid in early diagnosis and potentially improve outcomes in this group.
